# MVPA Analysis of Intertrial Phase Coherence of Neuromagnetic Responses to Words Reliably Classifies Multiple Levels of Language Processing in the Brain

**DOI:** 10.1523/ENEURO.0444-18.2019

**Published:** 2019-08-14

**Authors:** Mads Jensen, Rasha Hyder, Yury Shtyrov

**Affiliations:** 1 Center of Functionally Integrative Neuroscience (CFIN), Department of Clinical Medicine, Aarhus University, 8000 Aarhus, Denmark; 2 Laboratory of Behavioural Neurodynamics, St. Petersburg State University, St. Petersburg, 199034, Russia

**Keywords:** language, magnetoencephalography (MEG), multivariate pattern analysis (MVPA), oscillations, lexical access, semantics, morphosyntax

## Abstract

Neural processing of language is still among the most poorly understood functions of the human brain, whereas a need to objectively assess the neurocognitive status of the language function in a participant-friendly and noninvasive fashion arises in various situations. Here, we propose a solution for this based on a short task-free recording of MEG responses to a set of spoken linguistic contrasts. We used spoken stimuli that diverged lexically (words/pseudowords), semantically (action-related/abstract), or morphosyntactically (grammatically correct/ungrammatical). Based on beamformer source reconstruction we investigated intertrial phase coherence (ITPC) in five canonical bands (α, β, and low, medium, and high γ) using multivariate pattern analysis (MVPA). Using this approach, we could successfully classify brain responses to meaningful words from meaningless pseudowords, correct from incorrect syntax, as well as semantic differences. The best classification results indicated distributed patterns of activity dominated by core temporofrontal language circuits and complemented by other areas. They varied between the different neurolinguistic properties across frequency bands, with lexical processes classified predominantly by broad γ, semantic distinctions by α and β, and syntax by low γ feature patterns. Crucially, all types of processing commenced in a near-parallel fashion from ∼100 ms after the auditory information allowed for disambiguating the spoken input. This shows that individual neurolinguistic processes take place simultaneously and involve overlapping yet distinct neuronal networks that operate at different frequency bands. This brings further hope that brain imaging can be used to assess neurolinguistic processes objectively and noninvasively in a range of populations.

## Significance Statement

In an MEG study that was optimally designed to test several language features in a non-attend auditory paradigm, we found that, by analyzing cortical source-level intertrial phase coherence (ITPC) in five canonical bands (α, β, and low, medium, and high γ) with machine-learning classification tools [multivariate pattern analysis (MVPA)], we could successfully classify meaningful words from meaningless pseudowords, correct from incorrect syntax, and semantic differences between words, based on passive brain responses recorded in a task-free fashion. The results show different time courses for the different processes that involve different frequency bands. It is to our knowledge the first study to simultaneously map and objectively classify multiple neurolinguistics processes in a comparable manner across language features and frequency bands.

## Introduction

A neuropsychological assessment of the neurologic and/or cognitive status of a subject or patient is a routine required in a variety of situations. This typically involves elaborate behavioral tests aimed, for instance, at evaluating the extent of developmental disorders, assessing the level of neurodegenerative impairment, neurologic damage after a head injury, screening for hearing loss, etc. To perform such tests successfully, the subject, on the one hand, must typically have a reasonably clear understanding of what they have to do in a particular procedure; on the other hand, they must also be able to communicate their responses to a given task, by giving, e.g., a manual, facial or oral response. This raises the problem of uncooperative subjects, such as those suffering from neurologic/organic brain disorders or mental illnesses that cause patients to become unresponsive. A brain-damaged person may be unable to respond verbally because of a collateral lesion-related injury (such as akinetic mutism, paralysis, aphasia). A young child with a developmental disorder may be unable or unwilling to communicate their reaction overtly. A locked-in patient, although not necessarily unconscious, is unable to respond due to the total loss of their motor control function (Ragazzoni et al., 2000; Laureys et al., 2004). On the other hand, an undetected language impairment could have drastic consequences for (mis)diagnosis of language-unrelated disturbances (Majerus et al., 2009). A range of such situations is wide and they create a substantial challenge for diagnosis and assessment of performance, development or recovery in various groups. Clearly, techniques that could reveal the brain processing of language without relying on the individual’s overt behavior would be helpful in such cases.

Fast-paced development of non-invasive neuroimaging techniques in recent years has given hopes of assessing the brain status of various cognitive functions objectively, even when overt response may not be possible. Language is a complex phenomenon that, to make a coherent whole, requires multiple information types involving different specific features and properties that rapidly unfold in time with a fine temporal structure and remarkable speed (Friederici, 2002, 2011; Shtyrov, 2010). This, in turn, suggests that for the capturing of cerebral processing of linguistic information, a temporally-resolved neuroimaging method is needed that could faithfully track the dynamic neural activity during language comprehension (Hagoort, 2008). At present, two main techniques are able to provide such high temporal resolution: MEG and EEG, both capable of registering mass neural activity on a millisecond scale. Whereas the two methods are highly similar, MEG has a certain advantage in the ease of modeling underlying cortical activity, owing to unhindered spreading of magnetic fields (but not electric currents) through the head and skull tissues (Baillet et al., 2001).

A body of electrophysiological research done in EEG and MEG using various linguistic materials has provided a rich picture of linguistic processing in the brain. Perhaps the most well-known neurophysiological response to language stimuli is the so-called N400 (Kutas and Hillyard, 1980), usually seen as an index of (lexico)semantic processes and peaking at ∼400 ms. Syntactic (grammatical) processing has been associated with an early ELAN (early left anterior negativity) response reflecting the stimulus’ grammaticality from ∼100 ms (Neville et al., 1991; Friederici et al., 1993), as well as later frontal negativities (LAN) with longer latencies (Münte et al., 1998; Gunter et al., 2000) and the P600, a late positive shift (Osterhout et al., 1994).

Most of these responses (except, to an extent, ELAN) still require at least a degree of overt attention or even focused task performance from the subject. In terms of assessing linguistic processing in a more task-free fashion, a number of studies have attempted to use the so-called passive paradigms, in which the subjects are presented with linguistic contrasts without having to perform an overt task and are usually distracted from the auditory input by a video or another unrelated activity (Pulvermüller and Shtyrov, 2006; Näätänen et al., 2007). A series of studies using this approach established MEG/EEG correlates of automatic linguistic access including phonological, lexical, semantic, and syntactic levels of information processing (Shtyrov, 2010). These have shown that information-specific linguistic activations can be recorded noninvasively without an explicit task or even an instruction to focus on the speech input. For instance, meaningful native words show stronger activation in the core language system than meaningless acoustically similar pseudowords, which putatively indicates activation of word-specific memory traces (Pulvermüller et al., 2001). Furthermore, these activations show both semantic specificity (e.g., by involving motor cortex activation and deactivation when presenting action-related words; Hauk et al., 2008; Moseley et al., 2013; Shtyrov et al., 2014) and sensitivity to grammatical properties (ELAN-like activity in response to syntactic violations; Hasting et al., 2007).

Such studies of neurolinguistic processing typically focus on one single process (e.g., syntax or semantics) at a time. For this approach to be more practically-oriented, it would seem essential to develop a task-free paradigm that can assess multiple types of linguistic information processing in a single short participant-friendly session. Furthermore, previous studies have mostly investigated neurolinguistic processes using ERP/ERFs, that is, evoked activity in a rather narrow frequency band (typically 1–30 Hz), and it would thus appear advantageous to open up the frequency spectrum to maximize possibilities for registering brain reflections of language. Oscillations in different frequency bands have been shown to reflect multiple cognitive processes and are considered to be a vehicle for neural communication (Varela et al., 2001; Fries, 2005). Different frequency bands have been ascribed different functional roles in the neocortical information processing (Sauseng and Klimesch, 2008; Cannon et al., 2014), where higher bands could be related to local computations (Buzsáki and Silva, 2012; Buzsáki and Schomburg, 2015) and lower bands to longer-range connectivity (Von Stein and Sarnthein, 2000). That different neurolinguistic processes involve different frequency bands has been shown in a range of studies. For instance, Bastiaansen and Hagoort (2006) argued that retrieval of lexical information and unification of semantic and syntactic information are underpinned by different oscillatory networks, while Kösem and van Wassenhove (2017) suggested that θ oscillations (3–8 Hz) are related to acoustic properties. Teng et al. (2017) shows that humans can track spoken language dynamics at both θ and γ oscillations. Using electrocorticography (ECoG), Towle et al. (2008) have shown an increase in high γ (70–100 Hz) in relation to hearing a word compared to a tone. Luo and Poeppel (2007) showed that phase information in the oscillatory dynamics can track and discriminate spoken sentences. However, although the interest in time-frequency analysis of electrophysiological data has been steadily rising in recent years, language-related oscillatory dynamics still remains relatively unexplored, with most studies in the field focusing on evoked potentials/fields instead.

In an attempt to close the gap between these research strands and at the same time bridge this research with applied/clinical needs, we set out to combine these different approaches and designed a simple short paradigm that simultaneously includes lexical, semantic, and syntactic contrasts, to assess different levels of linguistic processing in a single session. This paradigm is tested here by recording MEG responses in a sample of healthy adult participants as a first step to establishing its applicability. The participants were presented with spoken stimuli, which were either meaningless pseudowords or meaningful words with different semantics (action-related verb vs concrete visual noun) and that either could be syntactically correct or included a morphosyntactic stem-affix violation. These were presented without any stimulus-related task, while the subjects’ attention was diverted away from the auditory stimulus stream.

Furthermore, in order not to restrict our results to narrow-band evoked activity, we analyzed activation in a wide range of frequency bands, from α to high γ. We focused on the phase part of the oscillatory activity, as phase synchrony has been theorized to be directly related to neural computations (Fries, 2005; Palva et al., 2005; Lopes da Silva, 2006; Varela et al., 2001). To quantify coordinated neural phase activity related to neurolinguistic processing, we determined phase synchrony by analyzing intertrial phase coherence (ITPC) of the MEG responses. ITPC is a measure of how aligned the phase angles over individual trials are. It therefore provides information about mass-synchronized neural activation (and in this sense is to a degree similar to ERP/ERF) without restricting it to a specific power peak or band. ITPC can inform us about the phase synchrony over trials; i.e., if ITPC is high, it means that the phases become aligned when performing a particular perceptual or cognitive computation. We investigated the ITPC in different frequency bands allowing for a comprehensive assessment of the neurolinguistic dynamics. To gain anatomic specificity, single-subject MRIs were used to model cortical source activity, and ITPC values were calculated in individual source space.

Given the vast amount of information about neural activity created by analyzing the ITPC across both time and frequency bands, we used multivariate pattern analysis (MVPA) for an unbiased statistical assessment of the data (Bzdok et al., 2018), where by “unbiased” we mean that that the researcher does not have to choose when and where to test for differences. MVPA (for more details, see Materials and Methods) is a machine learning technique based on predicting data rather than on parameter estimation (as used in traditional factorial analyses, e.g., ANOVA or *t* tests). The MVPA algorithm will first try to extract a pattern from a subset of the data, which can then be used to predict new, previously unseen data. This allows for a data-driven analysis without a priori hypotheses about spatial or temporal location of the signal of interest. For instance, we might allow the decoding algorithm to train itself on MEG recording trials of meaningful versus meaningless word form stimuli (i.e., find specific patterns of features in the data most valuable for detecting word-pseudoword differences) and then ask whether it can correctly predict the same distinction in a different data subset; this can be done both within and across subjects. Crucially, such an algorithm, if successful, may in principle be used to estimate data predictability in one subject (e.g., patient) from another one (e.g., healthy norm) and thus determine both normal functionality and functional abnormalities, or assess the same or different states of a particular processing system in the same individual.

In sum, we present here a short (<30 min) task-free paradigm in which auditory linguistic stimuli are presented to MEG subjects, without explicitly requiring their attention, and the resulting data are analyzed using MVPA-based machine learning algorithm to classify brain responses in different frequency bands as reflecting meaningful lexical input as well as its semantic and syntactic properties.

## Materials and Methods

### Participants

The experiment was conducted according to the principles of the Helsinki Declaration and was approved by Central Jutland Region Committee on Health Research Ethics. MEG data were acquired in seventeen healthy right-handed (handedness assessed using Oldfield, 1971) native speakers of Danish (age range 18–27 years, 12 females) with normal hearing and no record of neurologic impairments. All participants gave written consent before the start of the experiment and received payment for their participation.

### Stimuli

**Figure 1.**
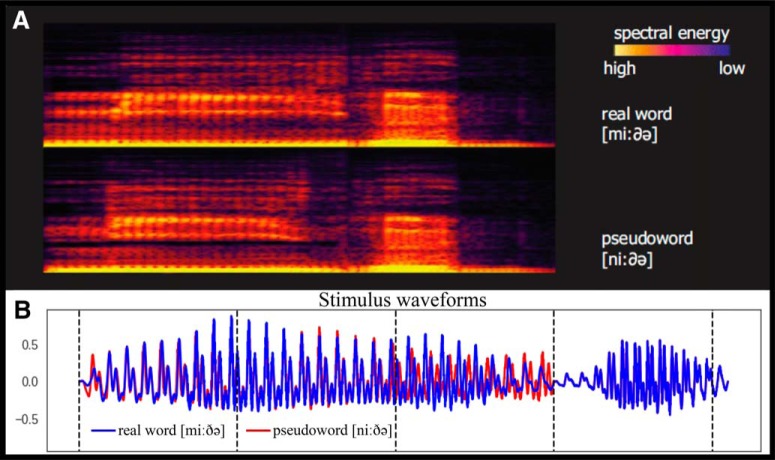
***A***, Examples of spectrograms of spoken stimuli used in the experiment (adapted from Gansonre et al., (2018)). ***B***, Examples of waveforms plotted on top of each other.

Since we wished to address a range of different neurolinguistic processes, those at lexical, semantic, and syntactic levels, we chose stimulus items which could enable us to contrast a combination of different linguistic phenomena while controlling for acoustic features (for examples of the stimuli used, see Fig. 1). To this end, we followed a previously suggested strategy (Gansonre et al., 2018) and selected a set of spoken Danish-language stimuli which (1) belonged to different lexical and semantic categories (action-related verb, abstract verb, object-related noun and meaningless pseudoword), (2) were close in terms of phonology so we could compare them directly with minimal acoustic/phonetic confounds, and which (3) could be modified morpho-syntactically in the exact same way and nonetheless exhibit different linguistic properties (i.e., grammatically correct vs incorrect) such that we could test the very same contrasts in different linguistic contexts.

These requirements led to the choice of four main base stimuli: bide ([biðə], *to bite*), gide ([giðə], *to bother*), mide ([miðə], *a mite*), *nide ([niðə], *pseudoword). These were presented as such. Note that they have identical CVCV phonological structure and only differ in the first consonant. The second syllable [ðə], which allows recognition of the lexical items, is the same across all items. To ensure that the full recognition of each particular word form in the restricted experimental context is only possible at the second syllable, we also included, in a 1:1ratio with all other stimuli, all four first syllables in isolation: [bið], [gið], [mið] and [nið]. These served as fillers to ensure identical acoustic divergence point across the four types, to be used for time-locking brain activity to, and were not analyzed as such.

The above quadruplet provided us with a way to address both lexical and semantic contrasts. By estimating the brain activity elicited by the same word-final syllable [ðə], we could compare, on the one hand, word versus meaningless pseudoword activation, putatively indicating lexical access, which we expected to find its reflection in an automatic activation of the core left temporo-frontal language system (Tyler and Marslen-Wilson, 2008). On the other hand, by comparing action versus non-action items we could address semantically-specific aspects of these activations. Previous EEG, MEG, and fMRI research has indicated automatic involvement of the brain’s motor system in the comprehension of action-related verbs (Pulvermüller, 2005; Pulvermüller and Fadiga, 2010); we therefore expected more pronounced centro-frontal activity for the action verb *bide*, but not the concrete noun *mide*.

Based on the above base forms, we produced further stimuli that included a balanced morphosyntactic contrast. We took advantage of Danish morphology and the fact that the morphemes *-(e)t* and *-(e)n* can be used to express the past participle of verbs and definiteness on common nouns. This enabled us to compare the inflected items based on their syntactic congruence or incongruence, e.g., *-n* in mide*n* vs. *gide*n*, and *-t* in gide*t* vs. *mide*t* (where * indicates a violation of the stem/affix syntactic agreement). Note that each of these pairs have identical codas (*t/n*) that lead to grammatical/morphosytactic violation in a counterbalanced fashion: each of them is correct in combination with one but not the other stem. These were presented, in equal proportion along with the other stimuli above, to make sure syntactic properties are only recognized at the very last consonant. To balance for these acoustic modifications, we also included similar items based on other forms (bide[n/t] and nide[n/t], all meaningless), which were used to make a balanced design, but not analyzed as such.

The stimuli were made based on a digital recording of a male native speaker of Danish in an anechoic chamber (recording bandwitdh: 44k Hz, 16 bit, stereo). The first and second syllables of four CVCV stimuli were recorded independently, to avoid possible coarticulation effects, and cross-spliced together, such that the second syllables were physically identical across all items. The second syllable commenced at 300 ms after the onset of the first one, and this was the earliest time [the so-called disambiguation point (DP)] when any lexical or semantic effects could be expected in the MEG data.

To produce the morphosyntactic items, ending in [t] or [n], we cross-spliced recordings of these two morphemes onto the four main stems, to obtain words either violating or respecting rules of Danish morphology such that the exact same phonemes completed syntactic or asyntactic forms in a counterbalanced fashion. These morphemes became distinct at 408 ms after the word onset, and this was therefore the earliest time any morphosyntactic contrasts could affect the brain responses.

The sounds were matched for loudness, with a 1.93-dB drop between the first and the second syllables so that our stimuli sounded as natural as possible and normalized to have identical power (measured as root-mean-square, RMS). All sound editing was done using Adobe Audition CS6 software (Adobe Inc.).

In sum, the stimulus set included four CV syllables, four CVCV stems, four CVCV+[t] and four CVCV+[n] forms, all strictly controlled for phonological and acoustic properties. These were combined, in a pseudorandom fashion, in a single auditory sequence ensuring that the stimuli’s lexical, semantic, and syntactic properties were available at stringently defined times.

### Procedure

The MEG recording was conducted in an electromagnetically shielded and acoustically attenuated room (Vacuum Schmelzer GmbH). During the recordings, participants were instructed to focus on watching a silent film and to pay no attention to the sounds. The auditory stimuli were controlled using Neurobehavioral Systems Presentation v16 (Neurobehavioral Systems) and presented through in-ear-tubes (Etymotic ER-30) binaurally at 50 dB above individual auditory threshold.

All sixteen stimuli were presented equiprobably in a single data acquisition session intermixed in a continuous manner, with 100 pseudorandom repetitions of each stimulus resulting in 1600 epochs in total. The interstimulus onset-to-onset interval (stimulus onset asynchrony, SOA), was fixed at 1000 ms, based on previous studies of automatic neurolinguistics processing using non-attend designs, which served as the starting point for the current paradigm. The total recording time was 28 min.

MEG data were acquired with an Elektra Neuromag Triux MEG setup (Elekta Neuromag Oy), with 102 magnetometers and 204 planar gradiometers; for eye movement and heartbeat artifact detection two bipolar EOG and one bipolar ECG recordings were taken. Cardinal landmarks and additional head points were digitized using a Polhemus FASTRAK setup (Polhemus). Data were recorded at 1000 Hz, a high pass filter of 0.1 Hz and low pass of 330 Hz were applied online. Head position and head movements were continuously tracked using four head position indicator coils (HPIs). The participants were lying still on a non-magnetic patient bed, with their head as close to the top of the helmet as possible, the MEG dewar being in supine position.

### Data preprocessing

All data were preprocessed using MNE-python version 0.16 software package (Gramfort et al., 2013). First, continuous data were bandpass filtered from 1 to 95 Hz, downsampled to 500 Hz, and epoched into single-trial epochs of 1000-ms duration, starting 100 ms before and ending 900 ms after stimulus onset. Bad channels were automatically detected and interpolated, epochs with excessive bad channels discarded and outlier trials removed using an automatic approach (as implemented in autoreject utility; for details, see Jas et al., 2017). On average per subject there were 23.24 bad channels (median: 32, SD: 10.93) and 12.9 (SD: 18.66) bad epochs. No signal-space separation transformation (SSS; also known as maxfiltering) was applied at any stage of the preprocessing. Thereby cleaned epoch data were bandpass-filtered into five frequency bands (Dalal et al., 2011): α within 8–12 Hz, β (13–30 Hz), γ-low (30–45 Hz), γ-medium (55–70 Hz), and γ-high (70–90 Hz).

### Source reconstruction

For each participant, a T1 and T2 structural MRIs were obtained using a Siemens Tim Trio 3T MR scanner. The images were segmented with separate surfaces created for the gray matter, inner skull and skin using SimNIBS utility (Thielscher et al., 2015). For each subject, individual three-layer boundary element model (BEM) was calculated together with individual forward models. A common template gray matter surface was created by averaging all of the study participants, using FreeSurfer software (Dale et al., 1999).

Source reconstruction was conducted using an LCMV beamformer (Van Veen et al., 1997) using planar gradiometer data and previously developed (Westner, 2017; Westner and Dalal, 2017) Hilbert beamformer. The decision to use only gradiometers was chosen as mixing channel types is not trivial due to magnetometers and planar gradiometers producing values of different scales; furthermore planar gradiometers are less sensitive to external magnetic sources and have a better signal-to-noise ratio compared to magnetometers (Hari et al., 1988). First, for each frequency band of interest (α, β, γ-low, γ-medium, and γ-high), the epochs were bandpass-filtered for each subject without subtracting the evoked signal from the single trials, as we were interested in investigating the complete information contained in the responses time locked to the auditory stimuli. Secondly, an adaptive filter was created by combining responses to all the stimuli in the paradigm, using a three-layer BEM and a cortically constraint source space. The adaptive filter was computed using a data covariance matrix based on all time points of the bandpass-filtered epochs; the covariance matrix was not regularized before inversion. Source orientation was optimized by using the orientation of maximum signal power (Sekihara and Nagarajan, 2008). Neural activity index (NAI; Sekihara and Nagarajan, 2008), which incorporates the weight normalization using a unit-gain beamformer, was selected as the output value. Third, after the adaptive filters were created, the single-trial epoched data were Hilbert-transformed and the adaptive filter was applied to the complex data, providing a source reconstruction of the Hilbert-transformed single-trial data. Lastly, we calculated ITPC of thereby obtained single-trial source space data. This was done for each time point and in each source space location independently using the equation below:

$$ITPC_{tf}=\left|n^{-1}\sum_{r=1}^{n}e^{ik_{tfr}}\right|,$$

where *n* is the number of trials, *e^ik^* provides a complex polar representation of the phase angle *k* on trial *r* for the time-frequency point *tf*, where frequency is the frequency band (for review, see Cohen, 2014, chapter 19, especially pp. 244–245). This resulted in a single ITPC time course for each point in the source space for each subject.

After the source space ITPC data were calculated for each subject, the individual data were morphed onto a common template surface (5124 vertices) created as the mean over all individual participants for data standardization across the group. Finally, the data were smoothed with a 10-ms rolling window mean in temporal dimension for each source independently.

### MVPA

For each participant, the morphed ITPC time series for each point in the source space was extracted based on the contrast in question. Common for all the MVPA was the classification over time, i.e., for each time point a classifier pipeline was applied giving a classification score over time. We used the entire cortical source space for the classification at each time point. As it was comprised of 5124 vertices, it gave 5124 features per time point.

The classifier pipeline was constructed in MNE-python using scikit-learn utility (Pedregosa et al., 2011) and composed of three steps. First, the features were standardized (z-scored); the standardization was done across all vertices in the source space at each time point independently using the training set and then applied to the test set. Second, feature selection was done using cross-validated Lasso model (stratified folds, *n* = 4) to create a sparse feature space that was adaptive for each time point, i.e., allowing for number of relevant features to be different for different time points. Lastly, a logistic regression (C = 1) was used to classify the two contrasts, and receiver operating characteristic area under the curve (ROC-AUC) was used as the classification score. The pipeline was applied across subjects, meaning that we obtained a decoding score over time for all participants and, hence, we only looked for effects that could hold across the subjects in the tested population.

MVPA can easily overfit data (i.e., become biased toward a specific response) and, to prevent that from happening, cross-validation was used for the pipeline. Simply put, cross-validation makes use of all the data by first splitting the data into two smaller data sets. One set, called the training set, is used for standardizing the features and fitting (training) the MVPA model. The other data set, called the test set, is then used to test accuracy of the fitted model (i.e., trained model) by trying to predict the class of the new label and, by comparing the prediction to the actual class of the test set, we can calculate the ROC-AUC for the model. By creating new training and test sets from the full data set, it is possible to use all the data for both training and testing (for more details and strategies for cross-validating brain imaging data, see Varoquaux et al., 2017).

All the steps in the pipeline were cross-validated with stratified folds (*n* = 5). Stratified folds imply that ratio of classes in the all the data are maintained in all the cross-validated folds; e.g., in the lexical condition there are three real words and one pseudoword for each participant, so in the stratified folds the ratio 3:1 would remain such that even when shuffled there will always be a 3:1 ratio. For the semantic condition and morphosyntax comparison the ratio was 1:1, i.e., 50%. We had 17 participants in the study and it was, therefore, impossible to have an equal number in each of the cross-validation folds and to make cross-validation splits that have the exact same number of participants in all folds and keep the ratio the same. However, by using stratified folds we kept the balance between the folds as equal as possible.

To test for statistical significance of the classification we used permutation tests (Ojala and Garriga, 2010). A permutation test was performed for each time point independently. First, the arrangement of the labels was shuffled, e.g., action verb and object noun in the semantic condition, such that the data might be from an action verb but the label tell the MVPA algorithm that it is an object noun. By repeatedly shuffling the labels and running the classification (*n* = 2000), we can build a null distribution of random ROC-AUC scores. This null distribution is interpreted as the distribution of what the ROC-AUC score could be just by chance. Hence, we can then assess the classification score we had from the real labels by comparing to the null distribution. If number of random scores that are better than the actual classification score is less than or equal 5%, the classification score is said to be better than chance, where 5% is the α level chosen.

To optimize the computational time, only classification scores that surpassed the threshold (calculated, for each frequency band independently, as the mean score of the baseline plus 1.5 SDs of the baseline) were tested for statistical significance. So, for each time point where the ROC-AUC score was above the threshold we made a permutation test (*n* = 2000). As the minimum *p* value that can be obtained with a permutation test is dependent on the number of permutations run, the *p* value is defined as *p*_u_ = $\frac{\left(b+1\right)}{\left(m+1\right)}$, where *b* is the number of times the permutation was equal or more extreme then the observed classification value and *m* is the number of permutations, and 1 is added to *b* and to *m* to ensure that the estimated *p* value is not zero and to avoid division by zero (Phipson and Smyth, 2010; Puolivali et al., 2018). So, the *p* value in a permutation test does not behave in the same way as a *p* values in a parametric test, it is rather a description of observed probability that the given classifier is better than chance. Since we perform a series of permutations, i.e., one for each time point, we need to consider the multiple comparison problem, i.e., the problem that some tests will be significant purely by chance and not be a true effect. We opted to apply cluster threshold correction, i.e., there needs to be a continuous range of significant time points (at least 10 ms or longer) to accept it as a non-random effect. The 10-ms threshold is based on previous language and auditory research (Hagoort and Brown, 2000; Wang et al., 2012; Edmonds and Krumbholz, 2014), typically using 10 ms as the shortest bin duration to test. It should be noted that only two of the thirteen clusters reported as significant at 10 ms, the rest are longer, ranging from 12 to 42 ms.

For each linguistic contrast we fitted the classification pipeline independently. For the lexical contrast, we tested whether we could classify lexical features, i.e., if the participant heard a real word versus an acoustically similar pseudoword (see above, Stimuli). In the semantic contrast, we tested classification of the action verb versus object noun. In the syntactic contrast, we tested whether we could correctly classify ungrammatical versus grammatically correct items (irrespective of their acoustic features, i.e., *midet, *giden vs *miden*, *gidet*). Each contrast was tested from the relevant DP (300 ms for lexis and semantics and 408 ms for morphosyntax) through the end of the epoch. Latencies reported in Results below are measured relative to DP.

## Results

### Lexical contrast

**Figure 2.**
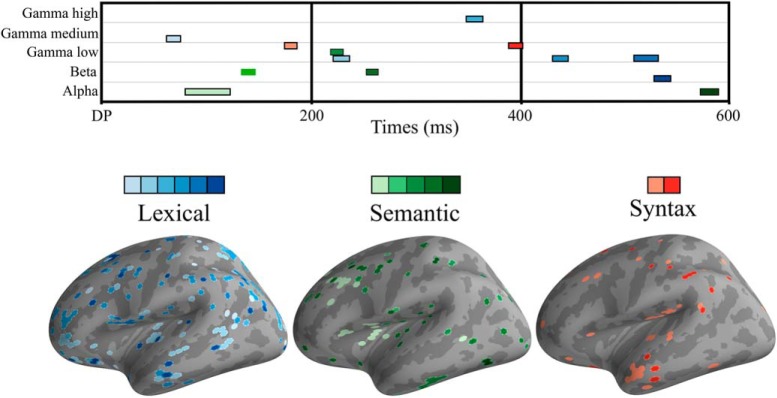
Top, Heatmap of significant clusters across three linguistic contrasts, five frequency bands, and time. Lexical condition in blue colors, semantic in green, and syntax in red. Bottom, Surface topography of significant effects. For all conditions, colors go from lighter to darker as latency becomes longer.

**Figure 3.**
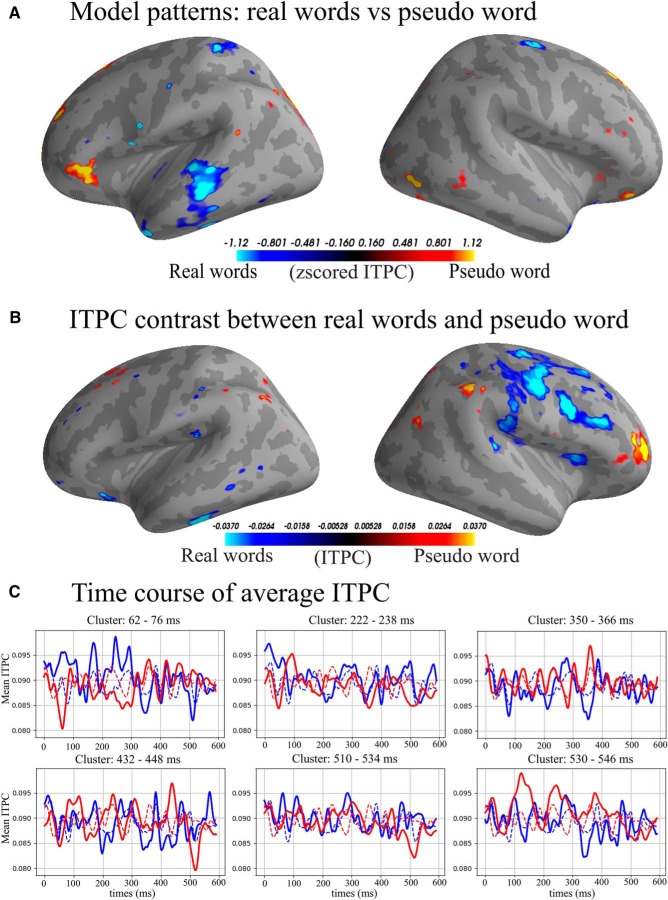
***A***, Model patterns: interpreting coefficients of a machine learning model is not trivial and a high coefficient value does not necessitate a high signal value in the MEG data (for details, see Haufe et al., 2014). “Model patterns” are a way to highlight the signal in a neurophysiological sensible way that is directly interpretable compared to the raw coefficients (Haufe et al., 2014). We show top and bottom 5% of the patterns in the γ-low band from 222 to 238 ms. Blue colors are areas of activation able to predict real words and yellow/red are areas used to predict pseudo word. ***B***, Average top and bottom 5% of ITPC difference; blue colors indicate higher ITPC for real words and yellow/red colors indicate higher ITPC for pseudo word γ-low band from 222 to 238 ms. ***C***, Average ITPC over time; solid lines are the average of the selected features, dashed lines are the average of all vertices in the source space. Time 0 is the divergence point, when stimuli could be recognized from the available acoustic information.

**Figure 4.**
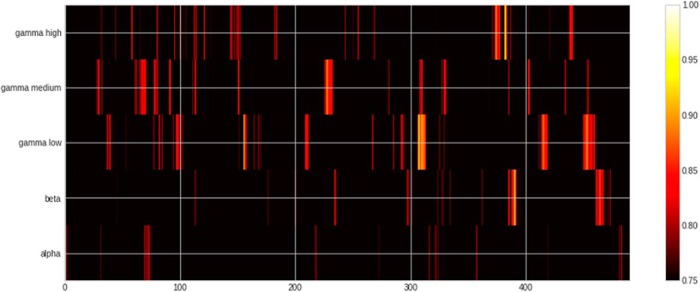
Heatmap of ROC-AUC scores for all bands in lexical condition. Note that chance in this condition is 75%. Time is relative to DP.

For the lexical contrast (Figs. 2–4; Table 1), we found that it was possible to significantly decode words versus pseudowords in the β, γ-low, γ-medium, and γ-high bands. The earliest significant decoding was achieved in the γ-medium band already at 62–76 ms after the DP. The pattern of classifier-selected features in the left hemisphere included the frontal lobe (BA 44, border of BA 6/BA 8, BA 4), parietal BA 7, junction of BA 7/39, and BA 39/19. In the right hemisphere, features in temporal lobe BA 22 and frontal BA 9, BA4 were selected. The highest ROC-AUC score was in the γ-low band at 224 ms after DP (ROC-AUC: 94.35%, SD: 4.5%). The cluster including this AUC-ROC peak spanned from 222 to 238 ms and involved a broad pattern of features including, in the left hemisphere, temporal lobe (BA 22), frontal areas (BA 11, border of BA10/47, BA44 dorsal/posterior), and parietal areas (BA 40, BA 7 as well as BA 3/1/2). In the right hemisphere, it included the temporal lobe (BA 22, BA 23, BA 43), frontal areas (BA 11, border of BA 9/10/46, posterior dorsal BA44), and parietal areas (BA 39, BA 40, BA 7, BA 1/2). No significant classification results were obtained in the α band.

**Table 1.** Table of significant clusters in the lexical condition sorted by time from the divergence point

Lexical								
Band	Peak (%)	Peak SD (%)	Peak time (ms)	Cluster start (ms)	Cluster end (ms)	Cluster length (ms)	Cluster mean (%)	Cluster SD (%)
γ-Medium	88.53	6.43	66	62	76	14	83.20	4.57
γ-Low	94.35	4.50	224	222	238	16	85.02	9.68
γ-High	87.88	9.29	358	350	366	16	80.19	4.95
γ-Low	87.65	11.08	440	432	448	16	81.34	4.99
γ-Low	87.71	8.83	516	510	534	24	81.34	4.36
β	85.97	14.95	538	530	546	16	80.25	4.32

Peak is highest ROC-AUC scores of the cluster. Peak SD is the standard deviation (SD) of cross-validation folds for the peak ROC-AUC score. Peak time is the time of the peak from DP. Cluster start is the start time of the cluster from DP. Cluster end is the end time of the cluster from DP. Cluster length is the length of the cluster. Cluster mean is the mean ROC-AUC score of the cluster. Cluster SD is the SD of the cluster mean across cross-validation folds.

### Semantic contrast

**Figure 5.**
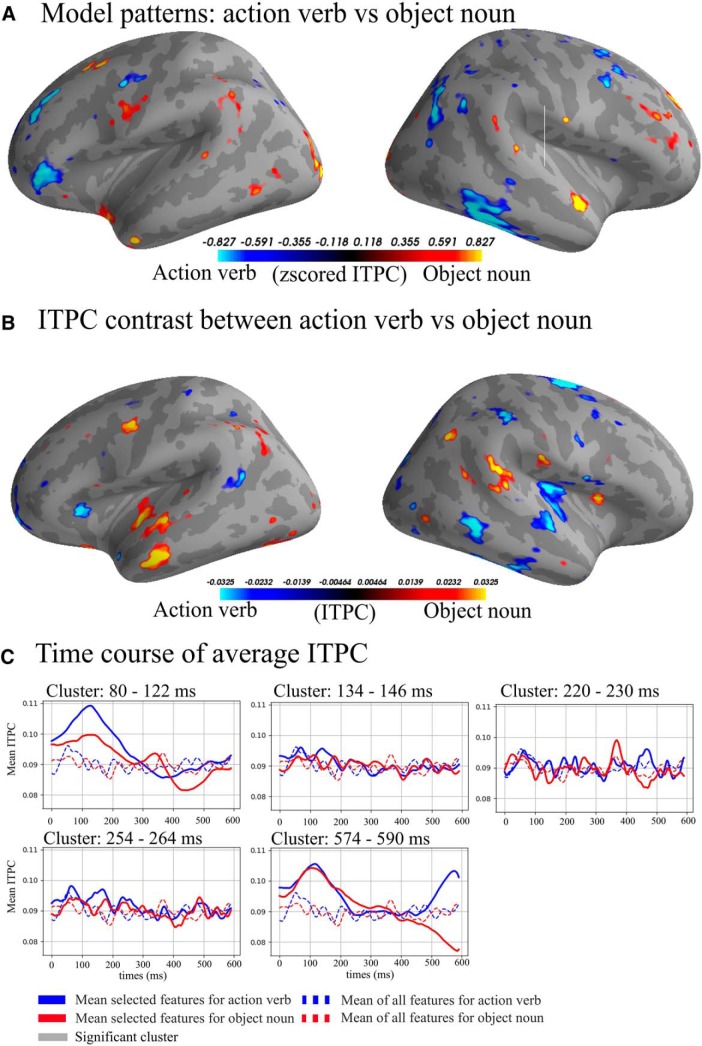
***A***, Model patterns (see also Fig. 4 legend): top and bottom 5% of the patterns in the the α band from 80 to 122 ms. Blue colors are areas used to predict action verb and yellow/red are areas used to predict object noun. ***B***, Average top and bottom 5% of ITPC difference, blue colors indicating higher ITPC for action verb and yellow/red indicating higher ITPC for object noun from 80 to 122 ms. ***C***, Average ITPC over time, solid lines are the average of the selected features, dashed lines are the average of all vertices in the source space. Time 0 is the divergence point, when stimuli could be recognized from the available acoustic information.

**Figure 6.**
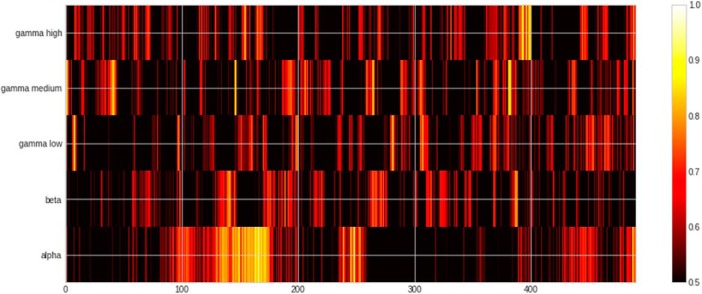
Heatmap of ROC-AUC scores for all bands in semantic condition.

In the semantic contrast (Figs. 2, 5, 6; Table 2), the peak classification score was found in the α band at 106 ms after DP (ROC-AUC: 91.11%, SD: 12.96%). The cluster including the peak ROC-AUC was between 80 and 122 ms after the onset of the second syllable disambiguating the semantics of the particular form. The cluster comprised a pattern of features including both temporal (BA 22) and frontal areas (BA 9, BA 44, BA 45, BA46) of the left hemisphere. In the right hemisphere, in addition to the temporal (BA 21, BA 22, BA 38, BA 42) and the frontal lobe (BA 6, BA 9, BA 11, BA 44), it also involved parietal areas (BA 7 and BA 2).

**Table 2.** Table of significant clusters in the semantic condition sorted by time from the divergence point

Semantics								
Band	Peak (%)	Peak SD (%)	Peak time (ms)	Cluster start (ms)	Cluster end (ms)	Cluster length (ms)	Cluster mean (%)	Cluster SD (%)
α	91.11	12.96	106	80	122	42	68.21	9.77
β	75.00	19.08	138	134	146	12	69.62	5.25
γ-Low	84.58	9.01	224	220	230	10	71.85	7.06
β	70.00	13.43	256	254	264	10	66.53	3.37
α	85.83	3.74	584	574	590	16	71.71	7.62

See the legend of Table 1 for an explanation of the columns.

### (Morpho)syntactic contrast

**Figure 7.**
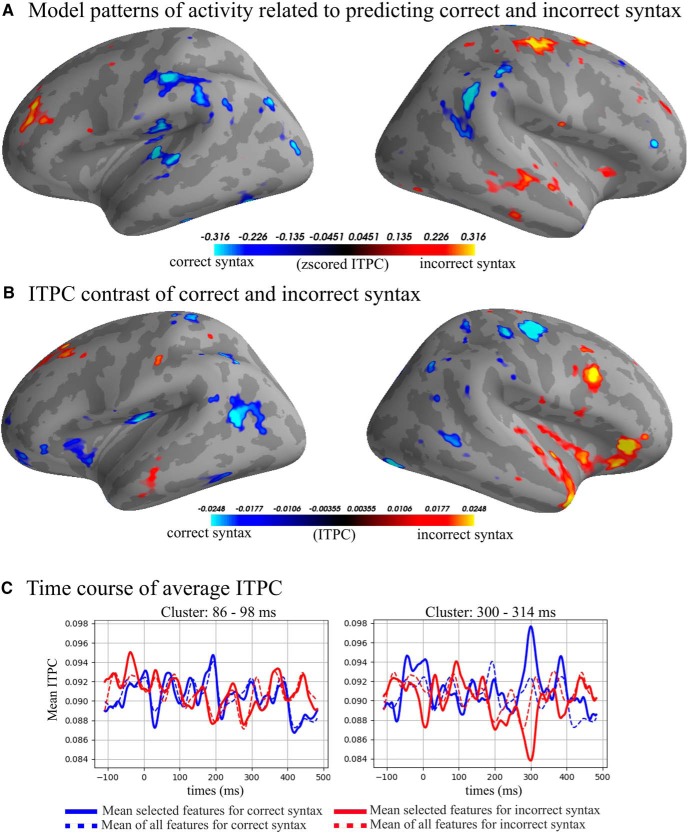
***A***, Model patterns: top and bottom 5% of the patterns in the γ-low band from 84 to 98 ms. Blue colors are areas used to predict correct syntax and yellow/red are areas used to predict incorrect syntax. ***B***, Average top and bottom 5% of ITPC difference. Blue colors indicate higher ITPC for correct syntax and yellow/red colors indicate higher ITPC γ-low band from 84 to 98 ms. ***C***, Average ITPC over time; solid lines are the average of the selected features, dashed lines are the average of all vertices in the source space. Time is relative to the divergence point.

**Figure 8.**
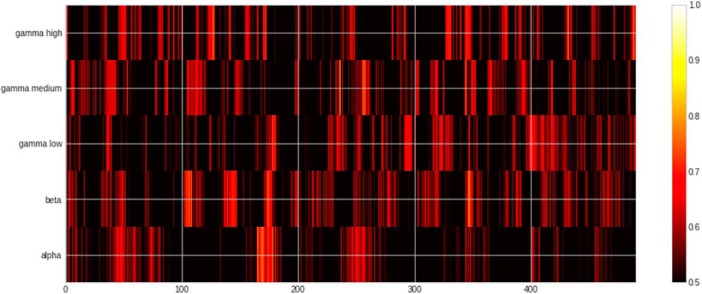
Heatmap of ROC-AUC scores for all bands in syntax condition.

The results of MVPA classification of grammatically correct versus incorrect inflections (Figs. 2, 7, 8; Table 3) showed the peak ROC-AUC score in the γ-low band at 90 ms after the syntactic DP (ROC-AUC: 69.22%, SD: 13.05%). The significant classification cluster on the left hemisphere included the temporal lobe (BA 21, BA 22, BA 37, BA 41), frontal areas (BA 4, BA 9 BA 10, BA 11), as well as parietal areas (BA 40/7/2), and occipital area BA 19. In the right hemisphere, it involved temporal (BA 21, 22, 38, 42), frontal (BA 44, 11, 4, 6, 9), parietal (BA 39, BA 40, 2, 1, 7), and occipital (BA 18, 9) areas.

**Table 3.** Table of significant clusters in the syntax condition sorted by time from the divergence point

Syntax								
Band	Peak (%)	Peak SD (%)	Peak time (ms)	Cluster start (ms)	Cluster end (ms)	Cluster length (ms)	Cluster mean (%)	Cluster SD (%)
γ-Low	69.22	13.05	90	85	98	12	62.57	9.16
γ-Low	68.36	11.57	302	300	314	14	64.04	5.10

See the legend of Table 1 for an explanation of the columns.

### Classification of amplitude data

We also tested *ad hoc* whether we could decode language properties using amplitude data. We only found significant clusters in semantic in the γ-medium band from 500 to 512 ms after DP. For the morphosyntactic condition, we found that we could classify correctly in the γ-high bands (from 114 to 128 ms after DP) and β band (from 248 to 262 ms after DP). As we were not able to decode all conditions successfully, we have left out the amplitude results from further discussion.

## Discussion

In this study, we suggested and tested a paradigm which, in the absence of focused attention on the auditory input or any explicit stimulus-focused behavioral task, addressed different levels of neurolinguistic information processing in the brain using a carefully crafted set of spoken stimuli with strictly controlled acoustic/psycholinguistic contrasts. We registered the brain’s activity elicited by these speech stimuli using high-density whole-head MEG set-up, and analyzed it using machine learning-based MVPA techniques applied to inter-rial phase coherence in a range of frequency bands, at the level of cortical sources calculated using individual MRIs. The results indicated that, by using this approach, we were able to successfully classify lexical, semantic, and morphosyntactic contrasts (for an overview of results, see Table 4). These effects were exhibited in different frequency bands and at different times. Below, we will briefly discuss these results in more detail.

**Table 4.** Table of peak scores for each bands and condition, dash (-) indicates no significant cluster

	Condition								
	Lexical condition			Semantic condition			Syntax condition		
Band	Peak score	Peak SD	Peak time	Peak score	Peak SD	Peak time	Peak score	Peak SD	Peak time
α	-	-	-	91.11	12.96	106	-	-	-
β	85.97	14.95	538	75.00	19.08	138	-	-	-
γ-Low	94.35	4.50	224	84.58	9.01	224	69.22	13.05	90
γ-Medium	88.53	6.43	66	-	-	-	-	-	-
γ-High	87.88	9.29	358	-	-	-	-	-	-

Peak score is highest ROC-AUC scores of the cluster. Peak SD is the SD of cross-validation folds for the peak ROC-AUC score. Peak time is the time of the peak from DP.

### Lexical contrast

In the lexical contrast, we found clusters of significant decoding performance (with a peak classification score of ∼94%) over the entire duration of the analysis epoch starting from ∼60 ms after the divergence point. These predominantly occurred in the γ range (including all three sub-bands tested) and were underpinned by activity in bilateral temporo-parietal and frontal clusters. This early onset of lexical effects here is in line with previous ERF and ERP studies that used similar non-attend auditory designs (Pulvermüller and Shtyrov, 2006; MacGregor et al., 2012; Shtyrov and Lenzen, 2017) and showed that the brain responds differently to meaningful words versus meaningless pseudoword stimuli from ∼50 to 80 ms after the acoustic input allows to identify the lexicality of stimuli, with additional lexicality-driven activity spanning across peaks until ∼400 ms. Such ERP/ERF results (Shtyrov et al., 2010; see also Shtyrov et al., 2005) have reported similar perisylvian configuration of bilateral source activations; importantly, here we find them for higher-frequency phase coherence values, not reported previously.

Increased activity in the γ band (quantified as spectral power or as event-related synchronization) has previously been linked to lexical access possibly underpinning synchronization of neural elements making up distributed word memory circuits (Lutzenberger et al., 1994; Pulvermüller et al., 1996; see also Tavabi et al., 2011). What our findings suggest is that this process may involve multiple synchronization steps expressed as phase resetting (an important mechanisms in information processing; Canavier, 2015) at different times, frequencies and neuroanatomical locations.

These previous studies have typically found a single power peak in low γ dynamics, whereas we here find a series of activations at frequencies up to 90 Hz, potentially reflecting the specific advantages of machine learning techniques in classifying distributed clusters of activity. We also found a single significant β cluster at the end of the epoch (>500 ms post-DP). This is fully in line with previous research highlighting the role of β oscillations in lexicosemantic storage and processing (Brennan et al., 2014; Bakker et al., 2015), although our data suggest that this β activity is secondary in relation to the almost immediate phase resetting in the γ band. Note that while a number of above studies have also identified θ band activity in relation to some aspects of speech processing (e.g., syllable tracking; Luo and Poeppel, 2007, 2012), we optimized our recording for time (with a view of potential applied use) that led to short baselines not suitable for θ analysis. There is, however, compelling evidence of a relation between θ and γ band activity (Canolty et al., 2006), and future research using similar paradigms could investigate whether the current ITPC findings may also have a counterpart in the θ range.

### Semantic contrast

The semantic contrast between the action verb and non-action noun has indicated activity from ∼100 ms after the divergence point in α, β and, to a smaller degree, in a lower γ range, with peak classification scores of ∼91, 75 and 85%, respectively. This mostly involved temporo-frontal cortices, largely overlapping with the core language systems, but importantly, indicated elevated frontal involvement, including clusters of activity in inferior-frontal (BA44, 45) and motor (BA6) areas, which is compatible with the motor system involvement in action word processing, posited previously (Pulvermüller et al., 2006). Previous EEG and MEG research into the brain basis of action-related semantics has found ERF and ERP correlates between 80 and 200 ms, indicating near-immediate and largely automated activation of the motor strip in action word comprehension (Shtyrov et al., 2004, 2014). In line with this previous research, the features indicated here by the semantic contrast did not include many in the temporal lobe but mostly in the inferior-frontal and prefrontal areas in the left and right hemispheres. While the timing of this activation was generally similar, and thus largely parallel, to the lexical processes above, the spectral composition was different, with significant ITPC findings in a lower frequency range. Changes in α and especially β power have previously been reported as related to single word semantics, including action word semantics in particular (Vukovic and Shtyrov, 2014; Bakker et al., 2015). What we show here is that the phase resetting likely linked to these changes can reliably classify words with different meaning. In oscillatory space our results are in line with those previously reported by Mamashli et al. (2019) who investigated functional connectivity across regions of interest and found activity in the α, β and low γ bands, while Haarmann and Cameron (2005) reported a change of coherence in the 10- to 14-Hz range when retaining a sentence.

### Syntax contrast

Successful classification of the morphosyntactic contrast was achieved in the γ-low range exclusively and was found to commence at ∼100 ms after the syntactic divergence point. While, in terms of the absolute stimulus timing, this difference was later than the lexical and semantic findings above, it is important to note the syntactic disambiguation in the stimulus itself was also possible at a later time, as it was determined by the final consonant (n/t). Thus, in terms of the relative timing, the syntactic properties appear to be assessed roughly in parallel to other tested features, overall in line with the view positing near-simultaneous onset of neurolinguistics processing of different information types (Marslen-Wilson, 1987; Hagoort, 2004). The timing is in line with the findings of ELAN literature that suggested syntactic parsing to sometimes start as early as 50 ms in an automatic fashion (Herrmann et al., 2011). Findings of links between γ band activity and syntactic parsing have been reported in the literature previously (Lewis et al., 2015), including morphosyntactic processing of the kind broadly similar to that required in comprehension of complex words used here (Levy et al., 2014). Importantly, we used a fully balanced set of contrasts, with highly matched word stimuli in which morphosyntactic (in)correctness was carried by physically the same phonemes, i.e., [n] and [t] equally employed in both sets. This activation was underpinned by broad temporo-frontal networks in both hemispheres, in line with previous literature (Hickok and Poeppel, 2007; Friederici, 2011; Bozic et al., 2015). We have also found activity in parietal and occipital areas, not commonly reported in syntax studies and therefore requiring validation in future research; notably, the values here do not reflect absolute activity (e.g., activity) as such but rather reliable classification of activation, however small it may be. Some studies investigating oscillatory neural activity in relation to syntactic processing have found that there is a link between α power and syntactic properties of phrases (Leiberg et al., 2006). While we do not find any similar activity in the α range for our morphosyntactic condition, that may be due to the use of single word stimuli with morphosyntactic modifications (rather than phrases with more elaborate syntax) in our paradigm.

Of the three linguistic contrasts tested, the morphosyntactic one had the lowest decoding percentage (peak at ∼69%). One possible reason for this is that it used two acoustically different stimuli, words ending in [n] and [t], with the grammatical correctness independent of the word ending. The different acoustic properties, later onset of [t] than [n] and different amplitude envelopes of the two, may have smeared the effect in time leading to poorer (but nevertheless significant) classification results. Future studies could use other contrasts and different languages to improve classification results.

### MVPA of MEG ITPC data as a tool for objective assessment of neurolinguistics processes

Previous literature has shown that passive paradigms can be used to investigate language processing (Shtyrov et al., 2012; Gansonre et al., 2018), an important first step toward assessing participants and patient groups that have difficulties responding verbally or in other behavioral ways. However, the next necessary step is to optimize the analysis of data obtained in such paradigms. Important issues arise when trying to automate MEG analysis both at preprocessing stage and at the level of identifying significant effects and differences either within or between groups. What we present here is a tentative proposal to solve these issues. By using the automated data cleanup protocol combined with a single-trial beamformer reconstruction, we reduce the number of manual steps needed; and further, by applying MVPA we avoid having to a priori select time and regions of interest (ROIs) for the statistical assessment of the difference between groups. The focus on oscillatory dynamics and ITPC provides us the ability to assess activity in different frequency bands simultaneously, resulting in a more detailed picture of the neural activity related to neurolinguistic processing. Previously, such analyses have been hampered by the number of tests that are needed to statistically evaluate the differences across groups/conditions. To solve this problem, we used MVPA, as this approach makes use of the powerful machine learning techniques capable of assessing the effects statistically in an unbiased fashion. So, by combining ITPC and MVPA we have the possibility to assess data in a detailed and yet exploratory manner.

Common to the effects across all conditions is that they last a relatively short time compared to the length of the trials. It is worth remembering that we are looking for clusters of time where we can decode a difference in the signals that are compared, which may help explain temporally sparse results. It is also worth noting that one cannot from a significant cluster alone conclude what exact processes underpin it. However, comparing the ITPC data and the patterns of the MVPA models together may provide an overall picture of the activity underlining the process at hand.

## Conclusions

Using a passive paradigm, we probed several different neurolinguistic properties. Separating the data into different frequency bands and looking at ITPC, we found that, by using MVPA, we could classify lexical, semantic, and syntactic information processing. The best classification results varied between the different neurolinguistic properties both in time and, importantly, frequency bands, with lexical processes classified predominantly by broad γ, semantic distinctions by α and β, and morphosyntax by low γ feature patterns. Crucially, all types of processing commenced in a near-parallel fashion from ∼100 ms after the auditory information allowed for disambiguating the spoken input. This shows that individual neurolinguistic processes take place near-simultaneously and involve overlapping yet distinct neuronal networks that operate at different frequency bands. Further investigations are needed to understand the precise relation of the time courses, frequency bands, neuronal substrates and neurolinguistic properties, and to test the applicability of this approach to detecting linguistic anomalies in various populations.

## Synthesis

Reviewing Editor: Satu Palva, University of Helsinki Decisions are customarily a result of the Reviewing Editor and the peer reviewers coming together and discussing their recommendations until a consensus is reached. When revisions are invited, a fact-based synthesis statement explaining their decision and outlining what is needed to prepare a revision will be listed below. The following reviewer(s) agreed to reveal their identity: Simo Monto.

Synthesis

The authors have considered the concerns raised by the reviewers and the manuscript has considerably improved. However, there are still several open issues that should be resolved before publication is warranted.

Major points:

1) Source-wise phase reconstruction. The explanation of source reconstruction and phase extraction is incomplete. The authors have maintained the manuscript as it was (L320) although they write different explanation in their point-by-point reply. Please explain how and in which order the source reconstructions were made, on which data the beamformer weights were applied, which data were Hilbert-transformed, and how the phase was extracted. As the authors used NAI as index of source activity, they should confirm that the used orientation selection and normalization affords for accurate reconstruction of the source-wise phase.

2) Group-level statistics. The part explaining MVPA and statistics on single subject level has improved considerably. Yet, there is still only one mention of cross-subject statistics (L366). It is thus left entirely unclear how group-level statistics were done. Furthermore, although authors state in their rebuttal that multiple corrections problem has been treated, this is not mentioned in the revised manuscript. Also, the 10-ms continuity requirement is left unexplained - the authors should give the reasoning behind this parameter, or they should consider e.g. temporal cluster analysis to identify significant continuous blocks.

Minor points:

1) The source reconstruction related figures (Figs. 3a, 5a, 7a) are largely unclear, unexplained and unreferenced: it is not described how these figures were created, nor what they should tell the reader. The title “Model patterns” in Figs 3A. 5A and 7A is ambiguous and wrong units (“ITPC”) are used. Also references to Tables are inaccurate, please double-check.

2) Just to confirm, MaxFilter was not used as a pre-processing step?

3) The authors should discuss why magnetometer data were discarded (L313).

4) How were features z-scored (L344)? Across time, space frequency, all or selected ones? Were the test data normalized accordingly?

5) the formula for p-value (L389) is more like “1-p” value and should be maximised instead of minimized.

## Author Response

We thank the Reviewers and the Editor wholeheartedly for the time and effort they spent evaluating our revised contribution, for their positive assessment of our thorough revision work, and for their very insightful and most useful comments that have greatly helped us to further improve the quality of the manuscript. We have now carefully and thoroughly revised the manuscript according to this new set of reviews. Below, we address all points raised by these valued colleagues in detail (with full original comments quoted in italics). In the manuscript, all changes are marked in red bold for easier identification.

Major points:

1) Source-wise phase reconstruction. The explanation of source reconstruction and phase extraction is incomplete. The authors have maintained the manuscript as it was (L320) although they write different explanation in their point-by-point reply. Please explain how and in which order the source reconstructions were made, on which data the beamformer weights were applied, which data were Hilbert-transformed, and how the phase was extracted. As the authors used NAI as index of source activity, they should confirm that the used orientation selection and normalization affords for accurate reconstruction of the source-wise phase.

Thank you. We apologise for not providing sufficient information on the details on the source reconstruction, which was previously omitted for brevity; this has now been corrected. We have updated the manuscript to provide more details on the process as stepwise sequence. The key points are that for each participant the adaptive filter is calculated for the bandpass-filtered epochs and responses to all stimuli are used to create the filter. After the filter is created, the epochs are Hilbert-transformed, and then the adaptive filter is applied to the complex Hilbert-transformed data, ultimately providing a source reconstruction of the Hilbert-transformed single-trial data. The single-trial source space data was then used to calculate the Inter-trial Phase Coherence (ITPC). These procedures are now described in detail in the revised manuscript (see Methods, lines 314-341).

Regarding dipole orientation, these were selected using max-power algorithm, and Neural Activity Index (NAI, Sekihara & Nagarajan, 2008) was selected as output value. The max-power selects the orientation of maximum power for every source point, and NAI incorporates the weight normalisation using a unit-gain beamformer. These details have also been clarified in revision (see lines: 327-330)

2) Group-level statistics. The part explaining MVPA and statistics on single subject level has improved considerably. Yet, there is still only one mention of cross-subject statistics (L366). It is thus left entirely unclear how group-level statistics were done. Furthermore, although authors state in their rebuttal that multiple corrections problem has been treated, this is not mentioned in the revised manuscript. Also, the 10-ms continuity requirement is left unexplained - the authors should give the reasoning behind this parameter, or they should consider e.g. temporal cluster analysis to identify significant continuous blocks.

Thank you very much for acknowledging the improvements and for making suggestions for further clarifications. We have now added more details on the group-level statistics and procedures in the revised manuscript and hope it is now clear how the group level decoding was performed and that we are decoding across subjects (see lines: 364-366; 416-421).

Minor points:

1) The source reconstruction related figures (Figs. 3a, 5a, 7a) are largely unclear, unexplained and unreferenced: it is not described how these figures were created, nor what they should tell the reader. The title “Model patterns” in Figs 3A. 5A and 7A is ambiguous and wrong units (“ITPC”) are used. Also references to Tables are inaccurate, please double-check.

Thank you and our apologies for the lack of clarity. We have supplied new legends for Figures 3, 5, and 7 to better explain what the model pattern is and how to gain information from it. The units and references to Tables have also been checked.

2) Just to confirm, MaxFilter was not used as a pre-processing step?

Correct. No (t)SSS/MaxFilter was applied to any of the data, as that would remove ranks requiring regularisation of the LCMV beamformer. We have added this information explicitly in the Methods section (lines: 300-301)

3) The authors should discuss why magnetometer data were discarded (L313).

Mixing channel types (e.g. gradiometers and magnetometers) is not without complications and at least require whitening of the data. Beamformer source reconstruction is usually done on a single channel type and we chose planar gradiometers, as they are less sensitive to eye blinks and other artefacts due to their more local sensitivity. We have added the reasoning for selecting gradiometers the manuscript (lines: 314-319)

4) How were features z-scored (L344)? Across time, space frequency, all or selected ones? Were the test data normalized accordingly?

Features were standardised as a part of the cross-validation pipeline, such that transformation values (mean and standard deviation) were calculated on the training set and then applied on the test set. So, for each fold a new mean and standard deviation was calculated. As the pipeline was run on each frequency and time point independently the standardisation was too. It was done across space, i.e. all the vertices for each fold. We have provided explicit detailed of these procedure to the revised manuscript (lines 358-359)

5) the formula for p-value (L389) is more like “1-p” value and should be maximised instead of minimized.

Thank you, and our apologies if the wording in our description was confusing. We have now tried to make the respective phrasing less ambiguous. There is no minimization or maximization as such going on during the estimation of the p-values from a permutation test. The p-values come from comparing the true classification score with scores where the labels of the y-vector are shuffled and then a new classification score is calculated. This is done repeatedly (we used 2000 permutations) creating a random distribution that takes the actual data into account; by counting how many of the random classifications scores that are better or equal to the true classification score we get a p-value of the classifier, which is calculated as pu = ((b+1))/((m+1)) , where b is the number of times the permutation was equal or more extreme then the observed classification value and m is the number of permutations, where b is the number of times the permutation was equal or more extreme then the observed classification value and m is the number of permutations: both b and m are increased by 1 (b+1, m+1) to ensure that the estimated p-value is not zero and that no division by zero is possible. This follows the approach of Ojala & Garriga (2010). We have updated the manuscript to explain this in more detail (lines 405-410; 400-405)

Once again, we would like to thank all Reviewers and Editors for their time, efforts and work in evaluating our manuscript; their comments, suggestions and criticisms were instrumental in improving this paper further. We believe that we have been able to address all concerns raised by these valued colleagues and hope that the newly revised manuscript will be acceptable for publication.

References

Ojala, M., & Garriga, G. C. (2010). Permutation tests for studying classifier performance. The Journal of Machine Learning Research, 11, 1833-1863.

Sekihara, K., & Nagarajan, S. S. (2008). Adaptive Spatial Filters for Electromagnetic Brain Imaging. https://doi.org/10.1007/978-3-540-79370-0
